# Montelukast Use Decreases Cardiovascular Events in Asthmatics

**DOI:** 10.3389/fphar.2020.611561

**Published:** 2021-01-13

**Authors:** Malvina Hoxha, Calogero C. Tedesco, Silvana Quaglin, Visar Malaj, Linda Pustina, Valerie Capra, Jilly F. Evans, Angelo Sala, G. Enrico Rovati

**Affiliations:** ^1^ Department of Pharmaceutical Sciences, University of Milan, Milano, Italy; ^2^ Department for Chemical-Toxicological and Pharmacologicsal Evaluation of Drugs, Catholic University Our Lady of Good Counsel, Tirana, Albania; ^3^ Centro Cardiologico Monzino IRCCS, Milan, Italy; ^4^ Department of Industrial Engineering and Information, University of Pavia, Pavia, Italy; ^5^ Department of Economics, Faculty of Economics, University of Tirana, Tirana, Albania; ^6^ Ministry of Education and Sports, Tirana, Albania; ^7^ University of Pennsylvania School of Medicine, Philadelphia, PA, United States; ^8^ IBIM, Consiglio Nazionale Delle Ricerche, Palermo, Italy

**Keywords:** asthma, montelukast, inflammation, cardiovascular diseases, leukotriene receptors

## Abstract

Cysteinyl leukotrienes are proinflammatory mediators with a clinically established role in asthma and a human genetic and preclinical role in cardiovascular pathology. Given that cardiovascular disease has a critical inflammatory component, the aim of this work was to conduct an observational study to verify whether the use of a cysteinyl leukotriene antagonist, namely, montelukast, may protect asthmatic patients from a major cardiovascular event and, therefore, represent an innovative adjunct therapy to target an inflammatory component in cardiovascular disease. We performed an observational retrospective 3-year study on eight hundred adult asthmatic patients 18 years or older in Albania, equally distributed into two cohorts, exposed or nonexposed to montelukast usage, matched by age and gender according to information reported in the data collection. Patients with a previous history of myocardial infarction or ischemic stroke were excluded. In summary, 37 (4.6%) of the asthmatic patients, 32 nonexposed, and five exposed to montelukast suffered a major cardiovascular event during the 3-year observation period. All the cardiovascular events, in either group, occurred among patients with an increased cardiovascular risk. Our analyses demonstrate that, independent from gender, exposure to montelukast remained a significant protective factor for incident ischemic events (78% or 76% risk reduction depending on type of analysis). The event-free Kaplan–Meier survival curves confirmed the lower cardiovascular event incidence in patients exposed to montelukast. Our data suggest that there is a potential preventative role of montelukast for incident cardiac ischemic events in the older asthmatic population, indicating a comorbidity benefit of montelukast usage in asthmatics by targeting cysteinyl leukotriene-driven cardiac disease inflammation.

## Introduction

Inflammation is a physiological reaction that, when dysregulated, can become a pathological process associated with several diseases characterized by the release of mediators, including metabolites of arachidonic acid. Cysteinyl leukotrienes (cysteinyl-LTs), namely, LTC_4_, LTD_4_, and LTE_4_, are derived from arachidonic acid and are potent proinflammatory lipid mediators well-known to play an important role in asthma (Nicosia et al., 2001), and that have also been implicated in other inflammatory conditions, such as allergic rhinitis (AR), atopic dermatitis, and urticaria (Capra et al., 2007). Unlike many other inflammatory mediators, cysteinyl-LTs are not decreased by steroid use in asthmatics (Gyllfors et al., 2006) Additionally, cysteinyl-LT involvement has been hypothesized in several cardiovascular diseases (CVDs), such as acute myocardial infarction (MI), ischemic stroke (IS), atherosclerosis, aortic aneurysms, and intimal hyperplasia (Folco et al., 2000; Back, 2009; Poeckel and Funk, 2010; Capra et al., 2013). Increased intracoronary production of cysteinyl-LTs was detected in patients undergoing coronary angioplasty (Brezinski et al., 1992) and by systemic urinary LTE_4_ excretion in acute MI and ischemic heart disease patients (Carry et al., 1992; Allen et al., 1993; De Caterina et al., 2010). Several proteins in the 5-lipoxygenase pathway, including both CysLT receptors, were found in the arterial wall of patients at different stages of atherosclerosis (Allen et al., 1998; Lötzer et al., 2003; Spanbroek et al., 2003; Di Gennaro et al., 2010). Finally, besides the increase in cysteinyl-LTs concentration in CVD, a number of genetic studies also support a link between cysteinyl-LTs, their receptors, and CVD (Helgadottir et al., 2004; Iovannisci et al., 2007; Bevan et al., 2009; Freiberg et al., 2010).

In the 1990s, after the discovery of an important role for cysteinyl-LT in asthma, 5-lipoxygenase pathway inhibitors and CysLT_1_ receptor antagonists (LTRAs) were developed and are now widely used to treat asthma and other allergic conditions (Capra et al., 2006). In CVD, and particularly in atherosclerosis, there is a crucial inflammatory component (Ross, 1999; Back and Hansson, 2015). Given an important role for cysteinyl-LTs in modulating vascular tone and inflammation (Sala et al., 2000; Bäck, 2007; Back et al., 2014), LTRAs have been proposed for potential therapeutic use in such diseases (Funk, 2005; Capra et al., 2013).

The potent and selective CysLT_1_ receptor antagonist montelukast was first approved by the Food and Drug Administration to be used in different stages of asthma both in adults and children and later on also for the treatment of seasonal and perennial AR (Capra et al., 2006). In addition, in preclinical animal models, montelukast significantly reduces the formation of atherosclerotic plaques and intimal hyperplasia and reduces reactive oxygen species and apoptosis, demonstrating beneficial effects on endothelial cell function and myocardial remodeling (Capra et al., 2013; Hoxha et al., 2017). Furthermore, montelukast inhibited oxidized low-density lipoprotein-induced monocyte adhesion to endothelial cells, suggesting a preventative role in the early stages of atherosclerosis (Di et al., 2017). Montelukast is also protective against aorta dilatation, and it reduces aortic rupture and aneurism development in three independent animal models of abdominal aortic aneurysm (Di Gennaro et al., 2018).

Therefore, a large body of preclinical research has shown a role of LTRAs in general, and of montelukast in particular, in controlling and reducing CV risk (Funk, 2005; Hoxha et al., 2017). Indeed, asthmatic patients receiving montelukast have lower levels of CV disease-associated inflammatory biomarkers and lipid levels (Allayee et al., 2007). A recent nationwide cohort study on incident or recurrent ischemic events provided a first indication for a role of montelukast for secondary prevention of CVD (Ingelsson et al., 2012). In order to explore a potential CVD preventive role for montelukast, we performed a 3-year observational retrospective study including eight hundred asthmatic patients exposed or nonexposed to montelukast and assessed the efficacy of montelukast in prevention of a major CV event such as heart attack or IS.

## Materials and Methods

### Data Collection

To obtain a large sample of the Albanian asthmatic population, a number of allergists/pathologists across the country were enrolled to contribute to this study by providing data about their asthmatic subjects (older than 18 years) based on their patient register and medical history. The study was retrospective, and medical records of 400 asthmatic subjects receiving montelukast (10 mg daily) and 400 asthmatic subjects not receiving montelukast were evaluated (from January 1st, 2012, to December 31st, 2014). Asthmatic subjects (International Classification of Diseases (ICD-10 v:2016) code 493) were diagnosed on the basis of GINA (Global Initiative for Asthma) criteria (https://ginasthma.org) on objective evidence of variable airflow obstruction. As the number of patients taking montelukast was the limiting factor of the study, we considered as montelukast-exposed all of the patients taking the drug for a period of at least 3 months (maximum exposure was 2 years), excluding all subjects with a previous history of MI or IS, as reported in their medical records (ICD:MI-I21 or IS-I63). Three months of drug assumption was considered the minimum to be coherent with the Ingelsson study (Ingelsson et al., 2012). These patients were randomly matched with patients not receiving montelukast as an antiasthmatic drug primarily by gender and secondly by age, considering the difference in the reported incidence of CVDs between male and female and with increasing age. In order to identify a predisposition of the monitored patients to an increased CV risk for MI and IS, information was obtained on drug therapies for major chronic conditions such as hypertension, diabetes mellitus, dyslipidemia, cerebrovascular disease, anemia, arrhythmias, epilepsy, allergies, psychosis, ulcers, cancer, and depression. General information, such as age, gender, patient educational level, residence, and income, was also recorded. Systemic blood pressure, body mass index, smoking, obesity, or cholesterol levels were not available for the majority of the patients and therefore were not taken into account in our analysis. According to ICD, the two groups were finally classified for the presence or absence of MI or IS.

### Study Design

We calculated that, for a two-group survival analysis comparison over a total time of 3 years and 400 patients per group, we would be able to detect as significant an HR of 0.8 or lower with a type 1 error alpha = 0.05 and a power of 80%.

### Statistical Analysis

Median and interquartile range (Q1–Q3) was used to describe age distribution due to its nonnormality. Also, the nonparametric Wilcoxon test was used to assess the difference in age between the two groups of patients, exposed or nonexposed to montelukast. Pearson’s chi-square test for association was used to test the association of montelukast exposure with the qualitative variables. All the available variables were considered as potential predictors of a CV event. However, because some drugs were used by less than 10 patients, those were not considered in the subsequent analysis. Cox (proportional hazards) regression analysis was performed pooling IS and MI as target events. All the CV events occurred among patients taking antihypertensive, antiplatelet, or anticholesterol drugs, i.e., among patients at increased “CV risk.” For this reason, those variables could not be included in a classical Cox regression model, but rather we used both a propensity score (PS) matching using different calipers (0.4-01) and a Cox model adjusted for PS. PS method balances the covariates observed between subjects in the treatment study group and the control group, eliminating disparities, while caliper is the margin thickness requirement for matching the difference between the treated unit and its matching control unit. PS has been calculated using a logistic regression on the treatment variable (i.e., exposure to montelukast), considering age, gender, residence, education, income, as well as antihypertensive, antiplatelet, diuretic, hypoglycemic, antihypercholesterolemic, and antiandrogen drugs as potential predictors of using montelukast. Concerning qualitative ordinal variables with more than two levels, analyses were performed using both all levels and aggregating levels in order to obtain binary variables. MI or IS was considered as events for survival analysis. For every patient, the observation started on January 1st, 2012, and all patients have been observed up to 3 years (with no drop outs) or until the first event. Event-free Kaplan–Meier survival curves were drawn for major CV events, and the difference between these two curves was tested using the log-rank statistics. For all the analyses, *p*-values lower than 0.05 were considered statistically significant. All statistical analyses were performed using SAS 9.4 (SAS Institute, Cary, NC, United States).

## Results

Baseline characteristics and variable distribution of the patients in our observational retrospective three-year study, overall, and according to montelukast use are reported in Table 1. We first noticed that patients exposed to montelukast have a significantly lower event rate than unexposed patients, 1.25% and 8%, respectively. We then analyzed possible relationships between demographic features and montelukast usage in our group of asthmatics. A significant difference was observed in the age of patients exposed (median = 60, 46 Q1–68 Q3) compared to nonexposed to montelukast (median = 64, 55.75 Q1–70 Q3). There was also a difference in montelukast exposure between rural and urban patients. In rural zone, 77.7% (108/139) of our asthmatic groups were exposed to montelukast, compared to only 44.2% of urban patients (292/661). In asthmatic patients with a lower education, only 28.1% were exposed to montelukast (68/242), compared to 59.5% with mid- to high level education (332/558). However, income did not show a significant relationship with montelukast use. The use of additional drugs appeared to be very balanced between the two cohorts, exposed and nonexposed to montelukast, with the exception of antiplatelet (5% vs. 9.75%) and antitumoral drugs (0% vs. 1.75%).

**TABLE 1:** Characteristics of both montelukast-exposed and nonexposed patients.

	Total	Exposed (400)	Nonexposed (400)	*p*-value
Gender	368M/432F	184M/216F	184M/216F	0.504
Median age, Q1–Q3		60, 46–68	64, 55.75–70	<0.0001#
Events	37^a^ | 4.6%	5 | 1.25%	32 | 8%	<0.00001
Mean montelukast exposure		7.83 months		
Residence				
Rural	139 | 17%	108 | 27%	31 | 8%	<0.00001
Urban	661 | 83%	292 | 73%	369 | 92%	
Educational level				
Low	242 | 30%	68 | 17%	174| 43.5%	<0.00001
Mid + high	558 | 70%	332 | 83%	226 | 56.5%	
Income				
Low	371 | 46%	175 | 44%	196| 49%	0.08
Mid + high	429 | 54%	225 | 56%	204 | 51%	
Drug prescription				
Antihypertensive	399 | 49.9%	198 | 49.5%	201 | 50.25%	0.89
Antiplatelet	59 |7.37%	20 | 5%	39 | 9.75%	0.01
Diuretic	167 | 20.87%	84 | 21%	83 | 20.75%	0.99
Antipsychotic	3 | 0.37%	0	3 | 0.75%	0.25
Antiandrogens	16 | 2%	5 | 1.25%	11 | 2.75	0.21
Antithyroid	7 | 0.875	3 | 0.75%	4 | 1%	0.99
Anti-hypercholesterolemic	132 | 16.5%	58 | 14.5%	74 | 18.5%	0.15
Antihistamines	9 | 1.125%	4 | 1%	5 | 1.25%	0.99
Antipeptics	3 | 0.375%	0	3 | 0.75%	0.24
Antiarrythmics	4 | 0.5%	1 | 0.25%	3 | 0.75%	0.62
Antianemics	2 | 0.25%	0	2 | 0.5%	0.48
Hypoglycemics	49 | 6.125%	20 | 5%	29 | 7.25%	0.24
Antiepileptics	2 | 0.25%	0	0	0.48
Anticancer	7 | 0.875%	0	7 | 1.75%	0.022
Antibiotics	3 | 0.375%	0	3 | 0.75%	0.25
Antidepressants	1 | 0.125%	0	1 | 0.25	0.99

^a^ Two patients suffered from both MI and IS; #Wilcoxon test; all the other *p*-values refer to Pearson chi-square test for association.

**FIGURE 1:**
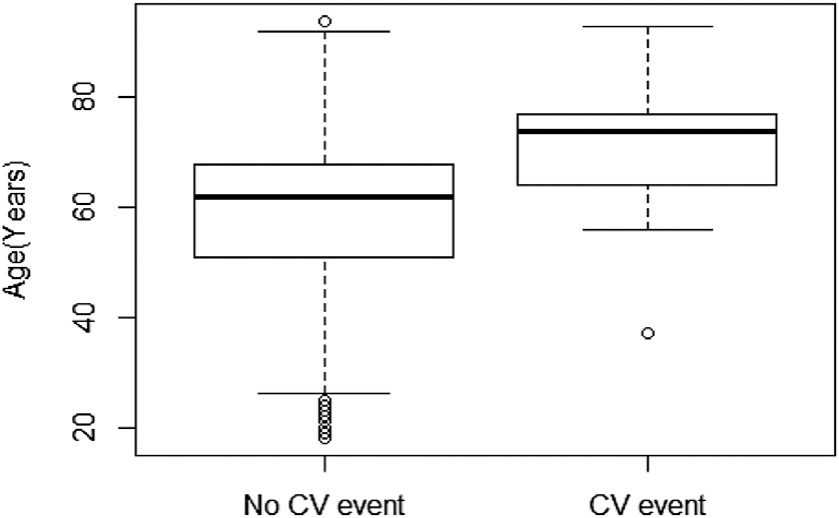
Box plot of age of patients without CV events (*n* = 763, median = 62, 51 Q1–68 Q3) vs. patients that suffered an ischemic event (*n* = 37, median = 74, 64 Q1–77 Q3). Statistical significance was calculated using the Wilcoxon test.

Our analyses showed that 37 (4.6%) of our asthmatic patients suffered a major CV event during the observation period. The overall incidence of an ischemic event in our asthmatic population (15.4 events per 1,000 patient-years) is similar to the expected rate in a general population [between 8 and 18.1 events, depending on level of blood pressure (Tajeu et al., 2017)]. However, in patients not exposed to montelukast, the incidence is considerably higher (26.7 events per 1,000 patient-years) than the incidence observed in the general nonasthmatic population. The CV event incidence in patients exposed to montelukast is 4.17 events per 1,000 patient-years, which is considerably lower than the 8 to 18.1 events expected in the general population (Tajeu et al., 2017). Our analysis of the incidence of CV events showed that the age of asthmatic patients that did not experience a CV event (median = 62) was significantly (Wilcoxon test, *p* < 0.00004) lower when compared to patients suffering a CV event (median = 74), with 12 years difference between the two groups (Figure 1). This is consistent with the well-known increase in CV events with increasing age.

We then performed PS matching using a COX model with different calipers. We focused on a caliper = 0.2 because it has been demonstrated to be optimal in many statistical settings (Austin, 2011) to look for predictors of a major CV event. PS matching resulted in assigning 269 patients in each group with a total number of 22 events. This analysis showed a highly significant (*p* = 0.0065) prevention of CV events (HR = 0.222) in the cohort exposed to montelukast. In the PS matching analysis, we lost some of the events (15 out of 37, Table 2), so we also used a Cox regression model adjusted for PS, which allowed us to take all the events into consideration (Table 3).

**TABLE 2:** Hazard ratio for CV events derived from propensity score matching.

Caliper	No. of patients in each group	No. of events	Hazard ratio	95% CI low	95% CI up	*p*-value
0.4	299	24	0.2	0.068	0.585	0.0033
0.3	284	23	0.211	0.072	0.619	0.0046
**0.2**	**269**	**22**	**0.222**	**0.075**	**0.657**	**0.0065**
0.1	245	20	0.176	0.052	0.602	0.0056

^a^ Values in bold are the values we decided to use.

**TABLE 3:** Hazard ratio for CV events derived from Cox regression using PS as a correction factor.

No. of patients in each group	No. of events	Hazard ratio	95% CI low	95% CI up	*p*-value
400 (all samples)	37	0.241	0.09	0.644	0.0046
59 (patients taking antiplatelet drug)	37	0.17	0.065	0.438	0.0003

Even though patients exposed to montelukast were overall slightly younger than those not exposed to the drug, montelukast remained a significant (*p* = 0.0046) protective factor (HR = 0.241) for CV events. In addition, as the use of antiplatelet drugs was significantly different between exposed and not exposed to montelukast (Table 1), namely, 5% vs. 9.75%, we took into consideration the antiplatelet bias by performing a Cox model adjusted for PS only on patients taking antiplatelet drugs. As shown in Table 3, the results are still statistically significant (*p* = 0.0003, HR = 0.17) and not statistically different (*p* = 0.87) from those considering the whole sample.

**FIGURE 2:**
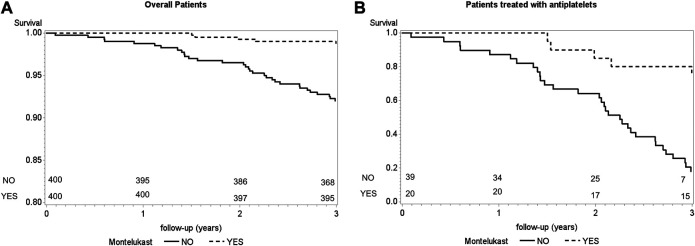
Event-free survival curves according to montelukast use [panel **(A)**, *n* = 800; panel **(B)**, *n* = 59]. Statistical significance was calculated using the log-rank test.

Figure 2 shows the event-free Kaplan–Meier survival curves of patients exposed or not exposed to montelukast. On the left panel, the total patient sample was considered, while on the right panel, only patients using antiplatelet drugs were considered. In both cases, the two curves were statistically different between montelukast-exposed and nonexposed asthmatics. As expected, the difference in survival probability was overall higher when limiting the analysis to patients treated with antiplatelets (log-rank test, *p* = <0.0001 and *p* = <0.0001).

## Discussion

Ischemic events such as MI and IS are among the leading causes of death worldwide. Atherosclerosis has been recognized to have a critical inflammatory component that is not well-treated by existing cardiac drugs (Ross, 1999; Rovati et al., 2010; Back and Hansson, 2015). Preclinical studies have shown a role for cysteinyl-LTs in a number of cardiac pathologies including atherosclerotic coronary arteries (Allen et al., 1998; Lötzer et al., 2003), while the 5-lipoxygenase activating protein (FLAP), an essential protein for cysteinyl-LT biosynthesis, has been linked to an increased risk of MI (Helgadottir et al., 2004; Hakonarson et al., 2005); we therefore hypothesized that LTRAs may have a role in CVD prevention (Hoxha et al., 2017). Asthmatic patients are more predisposed to CV events than the nonasthmatic population (Iribarren et al., 2012; Tattersall et al., 2015), and inflammation is a common feature of both asthma and atherosclerosis, so the use of a LTRA in asthmatic patients may have additional, comorbidity therapeutic benefit.

Our observational retrospective study in a homogenous population of eight hundred asthmatic adults in Albania has demonstrated a significant relationship between the use of montelukast and the reduction of a major ischemic CV event such as MI or IS even after correction for possible confounders. Our data indicate that, despite the limitations of the present study, montelukast may be of benefit in the prevention of CV disease in asthmatic patients. Analysis of montelukast usage in our sample population of asthmatic patients in Albania showed that patients treated with the drug were on average 4 years younger than those in the nonexposed montelukast group, however with a median age lower than 65 years for both groups. The clinical relevance of this modest difference between groups is uncertain, but given the large difference in the reported incidence of CVD between male and female (http://www.who.int/healthinfo/global_burden_disease/GBD_report_2004update_full.pdf), we used gender as primarily matching criteria and age as secondary criteria for inclusion.

As expected, the average age of patients incurring CV events, irrespective of montelukast treatment, was higher than those without CV events. This age-related increase in ischemic events in both groups indicates a substantial contribution of age to the CV risk, as one might expect in the aging population (Dhingra and Vasan, 2012). Both PS matching and PS-adjusted Cox analysis reveal that exposure to montelukast remains a significant protective factor for incident ischemic events, independently from gender. This observation extends to primary prevention of the results of Ingelsson and coauthors, who observed an effect of montelukast on recurrent MI in male and of recurrent IS in both genders, but no association of montelukast use with incident events (Ingelsson et al., 2012). Because asthma is recognized to be a possible confounder for the association of montelukast with CV diseases, limiting the sample to asthmatic subjects, as in our study, could have facilitated the detection of the effect of montelukast on incident events. All the events observed in our study occurred among patients taking antihypertensive, diuretic, antiplatelet, or anticholesterol drugs, namely, patients at increased CV risk of ischemic events.

Therefore, to further corroborate our findings and to exclude that use of antiplatelet drugs might bias our results, we also performed a PS-adjusted Cox analysis only on patients taking antiplatelet drugs. This again resulted in a statistically significant protective effect of montelukast not substantially different from that obtained in the overall sample. Given the difference between antiplatelet use between montelukast-exposed and nonexposed asthmatics, namely, 5% vs 9.75%, we showed that the event-free Kaplan–Meier survival curves for patients treated and nontreated with montelukast are statistically different, even if calculated only in patients taking antiplatelet drugs. Finally, our study may have underestimated the protective effect of montelukast since we considered as montelukast-exposed who had the CV event after stopping the drug. Indeed, only two out of five events in the exposed group occurred during montelukast treatment.

We acknowledge that there are a number of limitations of this study, such as the relatively limited number of events observed. However, our cohort is perfectly balanced for gender, a significant factor for CV diseases, and homogeneous with respect to asthma indication, which is recognized to be a confounding factor in the Ingelsson study (Ingelsson et al., 2012). Therefore, despite the number of events in the two studies being different, our 800 asthmatic samples might provide increased sensitivity in detecting the protective effect of montelukast. Other possible risk factors such as systemic blood pressure, body mass index, smoking, obesity, alcohol, or physical inactivity were not available for the majority of the patients, similar to the Ingelsson study (Ingelsson et al., 2012), and could not be included in our investigation. Conversely, the concomitant use of other drugs is well balanced between the two cohorts, with exception of the use of antiplatelet drugs. However, the results remained statistically significant when only asthmatic patients using antiplatelet were compared.

We suggest that a larger, case-control trial taking into consideration all CV risk factors is warranted by our data. Unfortunately, the montelukast patent life is expired, limiting the interest of the pharmaceutical industry to fund such a large, expensive CV trial. Thus, despite the impact that a definitive confirmation of these data might have on public health, only government-supported grants might provide enough funding for such a study.

In conclusion, our study highlights an additional benefit of leukotriene modifiers in asthmatics with the comorbidity of CVD. Targeting the cysteinyl-LT pathway, or indeed the total LT pathway with FLAP inhibitors (Pettersen et al., 2019), may be a strategy for primary prevention in populations at increased CV risk like asthmatics or secondary prevention in the general population. LTRAs in general and montelukast in particular are approved drugs used in clinical practice since almost two decades and are well tolerated (Bisgaard et al., 2009). The prevention of cysteinyl-LT-driven CV inflammation by the use of cysteinyl-LT antagonists or FLAP inhibitors may have an important benefit in reducing ischemic events that are among the leading causes of death in the developed world.

## Data Availability Statement

The original contributions presented in the study are included in the article/Supplementary Material, further inquiries can be directed to the corresponding authors.

## Ethics Statement

Ethical review and approval was not required for the study on human participants in accordance with the local legislation and institutional requirements. Written informed consent for participation was not required for this study in accordance with the national legislation and the institutional requirements.

## Author Contributions

MH: collected the patient data and participated in writing the manuscript. CT, SQ, and VM: performed the statistical analysis. LP: participated in collecting the data. VC: helped in designing the study and in discussing the data. JE and AS: major contributors in the discussion of results and the writing of the manuscript. GR: conceived the design and coordinated the study and was a major contributor in the discussion of the data and results and in the writing of the manuscript. All authors read and approved the final manuscript.

## Conflict of Interest

The authors declare that the research was conducted in the absence of any commercial or financial relationships that could be construed as a potential conflict of interest.

The handling Editor declared a shared affiliation, though no other collaboration, with one of the authors JE.

## Abbreviations

cysteinyl-LTs, Cysteinyl leukotrienes; AR, allergic rhinitis; CVDs, cardiovascular diseases; MI, myocardial infarction; IS, ischemic stroke; LTRAs, leukotriene receptor antagonists; GINA, Global Initiative for Asthma; PS, propensity score; HR, hazard ratio; FLAP, 5-lipoxygenase activating protein.

## References

[B1] Allayee H. Hartiala J. Lee W. Mehrabian M. Irvin C. G. Conti D. V. (2007). The effect of montelukast and low-dose theophylline on cardiovascular disease risk factors in asthmatics. Chest 132, 868–874. 10.1378/chest.07-0831 17646220

[B2] Allen S. Dashwood M. Morrison K. Yacoub M. (1998). Differential leukotriene constrictor responses in human atherosclerotic coronary arteries. Circulation 97, 2406–2413. 10.1161/01.cir.97.24.2406 9641692

[B3] Allen S. P. Sampson A. P. Piper P. J. Chester A. H. Ohri S. K. Yacoub M. H. (1993). Enhanced excretion of urinary leukotriene E4 in coronary artery disease and after coronary artery bypass surgery. Coron. Artery Dis. 4, 899–904. 10.1097/00019501-199310000-00009 8269196

[B4] Austin P. C. (2011). Optimal caliper widths for propensity-score matching when estimating differences in means and differences in proportions in observational studies. Pharmaceut. Stat. 10, 150–161. 10.1002/pst.433 PMC3120982 20925139

[B5] Bäck M. (2007). Leukotriene receptors: crucial components in vascular inflammation. ScientificWorldJournal 7, 1422–1439. 10.1100/tsw.2007.187 17767359 PMC5901331

[B6] Back M. (2009). Leukotriene signaling in atherosclerosis and ischemia. Cardiovasc. Drugs Ther. 23, 41–48. 10.1007/s10557-008-6140-9 18949546 PMC2663527

[B7] Back M. Hansson G. K. (2015). Anti-inflammatory therapies for atherosclerosis. Nat. Rev. Cardiol. 12, 199–211. 10.1038/nrcardio.2015.5 25666404

[B8] Back M. Powell W. S. Dahlén S. E. Drazen J. M. Evans J. F. Serhan C. N. (2014). Update on leukotriene, lipoxin and oxoeicosanoid receptors: IUPHAR Review 7. Br. J. Pharmacol. 171, 3551–3574. 10.1111/bph.12665 24588652 PMC4128057

[B9] Bevan S. Lorenz M. W. Sitzer M. Markus H. S. (2009). Genetic variation in the leukotriene pathway and carotid intima-media thickness: a 2-stage replication study. Stroke 40, 696–701. 10.1161/STROKEAHA.108.525733 19131661

[B10] Bisgaard H. Skoner D. Boza M. L. Tozzi C. A. Newcomb K. Reiss T. F. (2009). Safety and tolerability of montelukast in placebo-controlled pediatric studies and their open-label extensions. Pediatr. Pulmonol. 44, 568–579. 10.1002/ppul.21018 19449366

[B11] Brezinski D. A. Nesto R. W. Serhan C. N. (1992). Angioplasty triggers intracoronary leukotrienes and lipoxin A4. Impact of aspirin therapy. Circulation 86, 56–63. 10.1161/01.cir.86.1.56 1617790

[B12] Capra V. Ambrosio M. Riccioni G. Rovati G. E. (2006). Cysteinyl-leukotriene receptor antagonists: present situation and future opportunities. Curr. Med. Chem. 13, 3213–3226. 10.2174/092986706778742963 17168708

[B13] Capra V. Bäck M. Barbieri S. S. Camera M. Tremoli E. Rovati G. E. (2013). Eicosanoids and their drugs in cardiovascular diseases: focus on atherosclerosis and stroke. Med. Res. Rev. 33, 364–438. 10.1002/med.21251 22434418

[B14] Capra V. Thompson M. D. Sala A. Cole D. E. Folco G. Rovati G. E. (2007). Cysteinyl-leukotrienes and their receptors in asthma and other inflammatory diseases: critical update and emerging trends. Med. Res. Rev. 27, 469–527. 10.1002/med.20071 16894531

[B15] Carry M. Korley V. Willerson J. T. Weigelt L. Ford-Hutchinson A. W. Tagari P. (1992). Increased urinary leukotriene excretion in patients with cardiac ischemia. *In vivo* evidence for 5-lipoxygenase activation. Circulation 85, 230–236. 10.1161/01.cir.85.1.230 1309444

[B16] De Caterina R. Giannessi D. Lazzerini G. Bernini W. Sicari R. Cupelli F. (2010). Sulfido-peptide leukotrienes in coronary heart disease—relationship with disease instability and myocardial ischaemia. Eur. J. Clin. Invest. 40, 258–272. 10.1111/j.1365-2362.2010.02261.x 20415701

[B17] Dhingra R. Vasan R. S. (2012). Age as a risk factor. Med. Clin. 96, 87–91. 10.1016/j.mcna.2011.11.003 PMC3297980 22391253

[B18] Di X. Tang X. Di X. (2017). Montelukast inhibits oxidized low-density lipoproteins (ox-LDL) induced vascular endothelial attachment: an implication for the treatment of atherosclerosis. Biochem. Biophys. Res. Commun. 486, 58–62. 10.1016/j.bbrc.2017.02.125 28246014

[B19] Di Gennaro A. Araújo A. C. Busch A. Jin H. Wågsäter D. Vorkapic E. (2018). Cysteinyl leukotriene receptor 1 antagonism prevents experimental abdominal aortic aneurysm. Proc. Natl. Acad. Sci. U. S. A. 115, 1907–1912. 10.1073/pnas.1717906115 29432192 PMC5828611

[B20] Di Gennaro A. Wågsäter D. Mäyränpää M. I. Gabrielsen A. Swedenborg J. Hamsten A. (2010). Increased expression of leukotriene C4 synthase and predominant formation of cysteinyl-leukotrienes in human abdominal aortic aneurysm. Proc. Natl. Acad. Sci. U.S.A. 107, 21093–21097. 10.1073/pnas.1015166107 21078989 PMC3000261

[B21] Folco G. Rossoni G. Buccellati C. Berti F. Maclouf J. Sala A. (2000). Leukotrienes in cardiovascular diseases. Am. J. Respir. Crit. Care Med. 161, S112–S116. 10.1164/ajrccm.161.supplement_1.ltta-22 10673238

[B22] Freiberg J. J. Tybjaerg-Hansen A. Nordestgaard B. G. (2010). Novel mutations in leukotriene C4 synthase and risk of cardiovascular disease based on genotypes from 50,000 individuals. J. Thromb. Haemostasis 8, 1694–1701. 10.1111/j.1538-7836.2010.03903.x 20456754

[B23] Funk C. D. (2005). Leukotriene modifiers as potential therapeutics for cardiovascular disease. Nat. Rev. Drug Discov. 4, 664–672. 10.1038/nrd1796 16041318

[B24] Gyllfors P. Dahlén S. E. Kumlin M. Larsson K. Dahlén B. (2006). Bronchial responsiveness to leukotriene D4 is resistant to inhaled fluticasone propionate. J. Allergy Clin. Immunol. 118, 78–83. 10.1016/j.jaci.2006.03.040 16815141

[B25] Hakonarson H. Thorvaldsson S. Helgadottir A. Gudbjartsson D. Zink F. Andresdottir M. (2005). Effects of a 5-lipoxygenase-activating protein inhibitor on biomarkers associated with risk of myocardial infarction: a randomized trial. Jama. 293, 2245–2256. 10.1001/jama.293.18.2245 15886380

[B26] Helgadottir A. Manolescu A. Thorleifsson G. Gretarsdottir S. Jonsdottir H. Thorsteinsdottir U. (2004). The gene encoding 5-lipoxygenase activating protein confers risk of myocardial infarction and stroke. Nat. Genet. 36, 233–239. 10.1038/ng1311 14770184

[B27] Hoxha M. Rovati G. E. Cavanillas A. B. (2017). The leukotriene receptor antagonist montelukast and its possible role in the cardiovascular field. Eur. J. Clin. Pharmacol 73, 799–809. 10.1007/s00228-017-2242-2 28374082

[B28] Ingelsson E. Yin L. Bäck M. (2012). Nationwide cohort study of the leukotriene receptor antagonist montelukast and incident or recurrent cardiovascular disease. J. Allergy Clin. Immunol. 129, 702.e2 (11). 10.1016/j.jaci.2011.11.052 22244598

[B29] Iovannisci D. M. Lammer E. J. Steiner L. Cheng S. Mahoney L. T. Davis P. H. (2007). Association between a leukotriene C4 synthase gene promoter polymorphism and coronary artery calcium in young women: the Muscatine Study. Arterioscler. Thromb. Vasc. Biol. 27, 394–399. 10.1161/01.ATV.0000252680.72734.10 17110605

[B30] Iribarren C. Tolstykh I. V. Miller M. K. Sobel E. Eisner M. D. (2012). Adult asthma and risk of coronary heart disease, cerebrovascular disease, and heart failure: a prospective study of 2 matched cohorts. Am. J. Epidemiol. 176, 1014–1024. 10.1093/aje/kws181 23139248

[B31] Lötzer K. Spanbroek R. Hildner M. Urbach A. Heller R. Bretschneider E. (2003). Differential leukotriene receptor expression and calcium responses in endothelial cells and macrophages indicate 5-lipoxygenase-dependent circuits of inflammation and atherogenesis. Arterioscler. Thromb. Vasc. Biol. 23, e32-6. 10.1161/01.ATV.0000082690.23131.CB 12816882

[B32] Nicosia S. Capra V. Rovati G. E. (2001). Leukotrienes as mediators of asthma. Pulm. Pharmacol. Therapeut. 14, 3–19. 10.1006/pupt.2000.0262 11162414

[B33] Pettersen D. Broddefalk J. Emtenäs H. Hayes M. A. Lemurell M. Swanson M. (2019). Discovery and early clinical development of an inhibitor of 5-lipoxygenase activating protein (AZD5718) for treatment of coronary artery disease. J. Med. Chem. 62, 4312–4324. 10.1021/acs.jmedchem.8b02004 30869888

[B34] Poeckel D. Funk C. D. (2010). The 5-lipoxygenase/leukotriene pathway in preclinical models of cardiovascular disease. Cardiovasc. Res. 86, 243 10.1093/cvr/cvq016 20093252

[B35] Ross R. (1999). Atherosclerosis—an inflammatory disease. N. Engl. J. Med. 340, 115–126. 10.1056/NEJM199901143400207 9887164

[B36] Rovati G. E. Sala A. Capra V. Dahlén S. E. Folco G. (2010). Dual COXIB/TP antagonists: a possible new twist in NSAID pharmacology and cardiovascular risk. Trends Pharmacol. Sci. 31, 102–107. 10.1016/j.tips.2009.11.007 20117851

[B37] Sala A. Rossoni G. Berti F. Buccellati C. Bonazzi A. Maclouf J. (2000). Monoclonal anti-CD18 antibody prevents transcellular biosynthesis of cysteinyl leukotrienes *in vitro* and *in vivo* and protects against leukotriene-dependent increase in coronary vascular resistance and myocardial stiffness. Circulation 101, 1436–1440. 10.1161/01.cir.101.12.1436 10736289

[B38] Spanbroek R. Grabner R. Lotzer K. Hildner M. Urbach A. Ruhling K. (2003). Expanding expression of the 5-lipoxygenase pathway within the arterial wall during human atherogenesis. Proc. Natl. Acad. Sci. U.S.A. 100, 1238–1243. 10.1073/pnas.242716099 12552108 PMC298757

[B39] Tajeu G. S. Booth J. N. III Colantonio L. D. Gottesman R. F. Howard G. Lackland D. T. (2017). Incident cardiovascular disease among adults with blood pressure. Circulation 136, 798–812. 10.1161/CIRCULATIONAHA.117.027362 28634217 PMC5580500

[B40] Tattersall M. C. Guo M. Korcarz C. E. Gepner A. D. Kaufman J. D. Liu K. J. (2015). Asthma predicts cardiovascular disease events: the multi-ethnic study of atherosclerosis. Arterioscler. Thromb. Vasc. Biol. 35, 1520–1525. 10.1161/ATVBAHA.115.305452 25908767 PMC4441553

